# Unlocking the Beat: Dopamine and Eye Blink Response to Classical Music

**DOI:** 10.3390/neurosci4020014

**Published:** 2023-06-20

**Authors:** Leigh M. Riby, Sam K. Fenwick, Dimana Kardzhieva, Beth Allan, Deborah McGann

**Affiliations:** Department of Psychology, Northumbria University, Newcastle NE1 8ST, UK

**Keywords:** dopamine, eye blinks, emotion, mood, attention, music, classical music

## Abstract

The present study examined music-induced dopamine release, as measured by a proxy measure of spontaneous eye blinks. Specifically, we explored the effects of uplifting and sombre tones in different sections of Vivaldi’s *Four Seasons* to investigate the affective content of musical pieces within one composition. Seventeen participants listened to four concertos (Major modes: “Spring” and “Autumn”, Minor modes: “Summer” and “Winter”) and a silence condition while completing a three-stimulus odd-ball attention task. Electrooculograms were recorded from electrodes placed above and under the left eye. Self-reported arousal and music preference measures were also gathered during the testing session. In addition, the P3a Event-Related Potential (ERP) component was analysed as another potential index of dopamine function. Results revealed significant differences in the blink rates during music listening and silence, with the largest effect observed for the sad, melancholic “Winter” concerto. However, no significant correlation was found between blink rate and music preference or arousal. Furthermore, no reliable association was found between blink rate and the P3a ERP component, suggesting that these measures tap into different aspects of dopamine function. These findings contribute to understanding the link between dopamine and blink rate, particularly in response to classical music. Crucially, the study’s discovery that the “Winter” concerto, with its sorrowful tone, significantly increased the blink rate highlights the significance of sad music and perhaps the programmatic qualities of this concerto to induce a strong emotional response.

## 1. Introduction

Music is a source of joy that plays a central role in the human experience and positively impacts physical and mental health. It effectively interacts with the parasympathetic nervous system, treating somatic stress symptoms such as blood pressure, heart rate, muscle tension, and increasing oxygen saturation [1,2]. In the clinical realm, randomised controlled trials have demonstrated that music can lower plasma cortisol levels, increase the production of cytotoxic T cells, and thereby boost immune system function [3]. Laboratory-based experiments have also found that music can increase pain detection and tolerance thresholds [4]. Additionally, music has been found to alleviate both acute [5] and chronic pain [6] in patients with various conditions, including fibromyalgia, multiple sclerosis, and migraines. Music can even prevent chemotherapy-induced nausea and vomiting [7]). Listening to music for 15–30 min, 3–4 times weekly, can relieve exhaustion, lethargy, and anxiety [8]. Similarly, music can help patients with depression label, communicate, and manage their emotions, facilitating self-healing [9]. In the cognitive field, listening to music can mitigate the decline caused by sleep deprivation, as measured by mental rotation and selective attention tasks [10]. Earlier work on the cognitive enhancing properties of music has primarily focused on the Mozart effect [11]. Notably, the music-enhancing effects have also been associated with the release of dopamine, a hormone associated with enhanced pleasure, emotional arousal [12], reinforcement learning, episodic and working memory (e.g., [13]), and reward circuits in the brain [14].

The proposed connection between dorsal and ventral striatum dopamine release during music listening, which is associated with pleasurable experiences, is highly relevant to the current investigation [15]. Music-induced dopamine release in the brain’s reward centres can produce feelings of pleasure and euphoria, much like the effects of other pleasurable activities such as eating and engaging in sexual activity. In this study, we use spontaneous eye blink rate (EBR) as a non-invasive proxy measure of dopamine (DA) activity in the striatum, which is the primary input nuclei of the basal ganglia [16], to investigate this connection. Stevens [17] first proposed the relationship between EBR and DA after observing abnormal ocular responses, including increased EBR, in untreated schizophrenia patients, a condition characterised by dopamine dysregulation in the prefrontal cortex (reduction, negative symptoms) and mesolimbic system (excessive, positive symptoms). Subsequently, many studies have examined the link between EBR and DA in a variety of contexts, including schizophrenia (e.g., [18,19,20]), Parkinson’s disease [21], amyotrophic lateral sclerosis [22], and progressive supranuclear palsy [23], among others. Some studies have measured EBR with recreational drug use [24] and alcohol abuse [25], while others have intentionally manipulated DA levels in healthy animals and humans using pharmacological manipulations (e.g., dopamine D1 receptor agonist SKF-82958 in marmosets; [26]). Many of these studies found a significant positive correlation between DA levels and EBR [27], supporting its use as a proxy measure. However, there is conflicting evidence regarding whether this association is driven primarily by D1 or D2 receptors [28]. Regardless, heightened DA levels in the basal ganglia are believed to trigger activity in the trigeminal complex, a critical component of the spontaneous blink generator circuit, resulting in increased EBR [29].

In numerous studies of behaviour, cognition, and affect, psychologists have increasingly turned to EBR as a proxy measure for dopamine activity. For instance, Slagter, Georgopoulu, and Frank [30] investigated EBR during a probabilistic task that required participants to choose between two symbols, each with a different probability of producing positive or negative feedback. They found that, as predicted, low EBR was linked to greater learning from negative outcomes. However, contrary to prediction, high EBR was unrelated to better learning from positive outcomes. These data support the idea that EBR reflects levels of dopamine and suggests that EBR may be specifically related to D2 receptor function, given that the dopaminergic D2 pathway is important in avoidance learning. Akbari, Chermahini, and Hommel [31] explored the association between EBR and cognitive flexibility by having participants generate multiple uses for common household items. They discovered that moderate EBR was linked to the greatest cognitive flexibility, while low EBR/DA levels were associated with the inability to think divergently and high EBR/DA levels with excessive idea formation, distracting participants from their primary objective ([27,32]). In a study by Maffei and Angrilli [33], EBR was examined during five intervals of film clips that varied in emotional valence and level of arousal. They discovered that, overall, the main variable explaining EBR was emotional valence, as fear (unpleasant/high arousal) and sadness (unpleasant/low arousal) clips resulted in higher EBR compared with erotic (pleasant/high arousal) and compassionate (pleasant/low arousal) clips. In the present investigation, we used a similar approach by presenting musical excerpts that differ in mode, specifically major modes eliciting euphoria versus minor modes evoking melancholy during Vivaldi’s *Four Seasons*.

According to neuroimaging studies, there is a positive correlation between music and DA activity. For instance, Blood and Zatorre [34] used positron emission tomography (PET) to show that frisson, which is characterised by shivers and goosebumps, is associated with increased regional cerebral blood flow in the left ventral striatum and dorsomedial midbrain, areas involved in reward processing. Similarly, Salimpoor et al. [35] found that listening to pleasurable music led to increased DA activity in the mesolimbic reward system, including both dorsal and ventral striatum, as indicated by a decrease in [11C]raclopride binding potential. Ferreri et al. [12] also demonstrated that administering a DA agonist increased hedonic experience, as well as the perceived value of music and willingness to spend money on songs. Conversely, individuals given a DA antagonist reported reduced pleasure and motivation to spend money. Together, these findings suggest that it is reasonable to predict an increase in EBR whilst listening to music, which is linked to DA activity. However, this remains tentative in view of some conflicting findings on the relationship between EBR, musical enjoyment, and DA activity [36].

An alternative explanation for the changes in EBR while listening to music may not be solely attributed to music, but rather to internal versus external information processing. Smilek et al. [37] found that EBR significantly increased during instances of mind wandering during a reading task, positing that high EBR is indicative of a shift from external to internal processing. However, this conclusion is tentative, as they did not report EBR at rest, leaving the possibility that their findings indicate that EBR reduces whilst focusing on visual stimuli, rather than increasing when focusing on internal thoughts. Many studies suggest a negative correlation between EBR and visual attention, such as Cardona et al. [38], who found EBR reduced during visual processing to prevent loss of essential task information. Nevertheless, several studies support Smilek’s theory (e.g., [39]) suggesting that increased EBR is linked to mind wandering and the decoupling of attention from the external to the internal world. Therefore, we may expect EBR to increase while listening to music due to its strong links with mind-wandering, which could depend on the mood and familiarity of the music and the nature of the task. Taruffi et al. [40], Koelsch et al. [41], and Deil et al. [42] found that various forms of music can lead to mind-wandering, which is often self-focused and can concern topics such as sadness, love, and nature. Sad music is particularly associated with the increased frequency of mind-wandering, while heroic music is associated with positive, motivational thoughts. Familiar music is also less likely to elicit mind-wandering and can improve performance on cognitive tasks. 

### The Current Study

The focus of the current work was to examine the relationship between spontaneous eye blink rate (a proxy measure of dopamine levels) while listening to Vivaldi’s *Four Seasons*, with specific predictions regarding each major (“Spring”, “Autumn”) and minor (“Winter”, “Summer”) mode concertos. It was predicted that dopamine levels would increase while listening to music (compared with baseline), with the largest increase occurring during the “Spring” concerto, known for its uplifting and affective content [43]. However, it was not unreasonable to predict the alternative hypothesis that the “Winter” concerto would have a greater increase in eye blink rate than “Spring”. This prediction was based on the idea that sad music may elicit a stronger emotional response than cheerful music, leading to a greater physiological response, including an increase in spontaneous blink rate. While definitions of sad music vary greatly in the literature, commonly observed qualities are ‘gloom’, ‘depression’, and ‘quiet sorrow’ [44]. If we look further at subcategories of sadness, melancholic music has consistent traits such as minor mode, lower pitch, use of legato, and dark timbre [45,46]. Thus, “Winter”’s dramatic, sombre, and melancholic tone and its minor mode make it consistent with conveying melancholy and sadness. The primary analyses, therefore, will also consider the alternative hypothesis by comparing the blink rate across all concertos to the baseline. Since emotional arousal and music preference are important moderators in any physiological response to music, they were also examined [47]. We anticipated that greater preference for classical music would exhibit a greater reactivity (increase in blink rate) to the presented Vivaldi’s *Four Seasons*. Similarly, those who reported greater arousal levels would again display stronger physiological responses and increased eye blink rates. Finally, for completeness, we report how our data here correspond to the dopamine brain indices (P3a ERP component; See [48]) and cognitive measures (reaction time during a cognitive task) reported in our wider project of work reported elsewhere (e.g., [43]). Together, the study aims to provide the groundwork for future studies wishing to examine the relationship between eye blink rate, dopamine levels, and music listening and explore the impact of music preference and arousal on these effects.

## 2. Method

### 2.1. Participants

Seventeen adults participated in the study (Mean years of age = 21.1; SD = 4.2; women *n* = nine; see [43] for part one of this program of work). The Department of Psychology, Northumbria University Ethics Committee approved the study. Participants were invited to take part in the study via email and verbal invitation with a brief description of what the study would involve. All participants provided written consent. The inclusion criteria were being between 18 and 65 years of age and right-handed.

### 2.2. Design

Each of the 17 participants carried out a 3-stimulus odd-ball attention task to mimic the processing of task-relevant and irrelevant information (e.g., [48]) whilst listening to music in 12 blocks (4 Concertos: “Spring”, “Summer”, “Autumn”, “Winter” × 3 Movement Blocks: fast, slow, fast). The three movement blocks encompassed fast and energetic representations of each season, followed by serene interludes, culminating in another burst of energy. The odd-ball task was presented to the participant on three occasions in a silence control condition. The concertos and silence conditions were presented in a random order.

### 2.3. Materials

The electrophysiological recordings were gathered using the Biosemi acquisition hardware (https://biosemi.com/; accessed on 2 April 2023). Electrooculograms were gathered by placing electrodes above and below the left eye. The EEG data collection has been described elsewhere [43]. However, we report the P3a ERP component data for completeness. The P300 ERP is a positive brain response occurring around 300 milliseconds after a stimulus. The P3a subcomponent is specifically related to processing novel or unexpected stimuli, reflecting the brain’s automatic orienting response to salient events. Importantly, this subcomponent has been associated with dopamine function [49]. The electrodes on the scalp for these components were the Cz and Fz only, where previous research has identified where these are to be centred [48]. The EEG signals were digitised at a rate of 2048 per second with a recording epoch of 1200 ms, with a pre-stimulus interval of 200 ms. The response window to capture the P3a ERP component of interest was 250–450 ms. The average amplitude in this time window was the measure considered.

Two questionnaires were also used in the present study. Firstly, to assess the impact of self-reported arousal and mood during task performance, the Bond Lader Visual Analogue Scale [50] was given at regular intervals, at baseline and after each concerto and silence control condition. This scale consists of 16 items which measure subjective feelings of alertness, contentment, and calmness. The participant must mark, on a 100 mm line, the extent to which the described state reflects their mood at that moment in time. Finally, the STOMP questionnaire was given at the end of the experiment to assess the important moderator of music preference [51]. This questionnaire was scored to capture the preference for mellow, unpretentious, sophisticated, intense, and contemporary music types. Participants were asked to rate their preference for each genre on a scale from 1 (Strongly Dislike) to 7 (Strongly Like).

### 2.4. Procedure

Informed consent was first given after receiving a detailed brief regarding the nature of the study. Participants sat in front of the computer running the E-prime software (Version 2.0; Psychology Software Tools, Inc., Sharpsburg, PA, USA), with an obstructed view of the second computer running the Actiview604 (see https://biosemi.com/, accessed on 2 April 2023) software to record vertical electrooculograms from the left eye. The electrodes set up for each participant also included electroencephalograms to gather event-related potentials (the focus here was the P3a), with an approximately 25 min set up time. Participants then completed the 3-simulus odd-ball attention task during either music listening (Vivaldi’s *Four Seasons*; 4 concertos with 3 movements each, with 12 pieces in total) or the control silent condition (three blocks to mimic the music conditions) described above. The only instruction received was to respond as quickly as possible to the target stimuli, which were presented in a train of frequent standard stimuli. The standard frequent stimuli comprised a red circle (12.6 cm^2^) presented at regular intervals (110 items). During this time, infrequent target stimuli (green square, 16 cm^2^, 20 items) were presented, which required a response on a standard computer keyboard spacebar (the response times to these target stimuli were the behavioural measure of task performance gathered). In addition, a rare novel stimulus, a large blue square (256 cm^2^, 20 items), was also presented where no response was required. Participants were not informed of the novel stimulus presentation in the task instructions, and orienting attention to these stimuli was considered a good measure of novelty processing and dopamine function [48]. Each stimulus remained on the screen for 100 ms. The inter-stimulus interval ranged between 930 and 1030 ms. Before the first presentation of the task and after each of the conditions, the Bond Lader questionnaire was completed. Completing the STOMP music preference questionnaire was the final task given to the participants. On completion, the electrophysiological apparatus was removed, and a full debrief describing the purpose of the study was given.

## 3. Results

The first analysis considered blink rate (total count) differences across the four concertos and the silence condition. A five condition (“Spring”, “Summer”, “Autumn”, “Winter”, Silence) x three block (one, two, three) analysis of variance (ANOVA) was conducted on these data. There was a significant main effect of condition, F (4, 64) = 3.8, *p* = 0.007. The main effect of block and the interaction between block and condition were not significant. An exploration of the main effect of condition using the Least Significant Difference (LSD) procedure revealed significant differences between all the comparisons with “Winter”. This analysis, therefore, demonstrated a high blink rate for this concerto compared with all other conditions (“Winter” > Silence, *p* = 0.014; “Winter” > “Spring”, *p* = 0.02; “Winter” > “Summer”, *p* = 0.025; “Winter” > “Autumn”, *p* = 0.038). These data are displayed in Table 1.

The next analyses considered the important moderators of arousal (data gathered using the Bond Lader visual analogue scale) and music preference (data gathered using the STOMP music preference questionnaire). A series of Pearson correlations between blink rate and the three components of the Bond Lader questionnaire (Alert; Content; Calm) across the conditions (“Spring”, “Summer”, “Autumn”, “Winter”, Silent) were carried out. These relationships considered the arousal levels after each music piece, controlling for arousal measurement taken at baseline. Arousal was not reliably associated with blink rate. For the “Spring” condition, all correlations were non-significant (Alert, all ps > 0.64; Content, all ps > 0.16; Calm, all ps > 0.75). For the “Summer” condition, all correlations were non-significant (Alert, all ps > 0.78; Content, all ps > 0.08; Calm, all ps > 0.42). For the “Autumn” condition, Content was negatively related to blink rate in all three blocks (Block 1, r = −0.50, *p* = 0.046; Block 2, r = −0.54, *p* = 0.032; Block 3, r = −0.54, *p* = 0.031), but not for Alert (all ps > 0.28) or Calm (all ps > 0.85). For the “Winter” condition, all correlations were non-significant (Alert, all ps > 0.60; Content, all ps > 0.08; Calm, all ps > 0.10). For the Silence condition, all the correlations were non-significant (Alert, all ps > 0.28; Content, all ps > 0.80; Calm, all ps > 0.07). The relationships between blink rate and music preference were non-significant (all ps > 0.06).

For completeness, we also examined the relationship between behavioural performance (reaction time to hits during the odd-ball attention task), brain measures of dopamine and novelty processing (P3a ERP) as previously reported (Riby, 2013), and blink rates. For reaction time, there were no reliable relationships between blink rates (Silence, *p* > 0.16; “Spring”, *p* > 0.34; “Autumn”, *p* > 0.06; “Winter”, *p* > 0.05). For the “Summer” concerto, there was a significant relationship between reaction time in Block 2 and blink rate, r = −0.50, *p* = 0.043. Similarly, no consistent relationships existed between the P3a ERP and blink rate. For silence, Block 1, there was a significant correlation between the P3a (Cz scalp site) and blink rate, r = 0.51, *p* = 0.03. For “Winter”, Block 3, there was a significant correlation between the P3a (Cz scalp site) and blink rate, r = 0.50, *p* = 0.034. All other correlations were non-significant.

## 4. Discussion

The present study aimed to investigate the relationship between dopamine and music listening by examining the effects of different concertos from Vivaldi’s *Four Seasons* using spontaneous eye blinking as a proxy measure. The study specifically sought to elucidate the physiological correlates of emotional tone in the music, focusing on the uplifting, major modes (e.g., “Spring”) concertos and the melancholic, minor modes (e.g., “Winter”) concertos. Notably, using Vivaldi’s *Four Seasons* offered a unique opportunity to examine the effects of both sad and happy music within a single musical composition. The study also explored the potential moderating effects of music preference and arousal on the relationship between dopamine and music, given their relevance to music and cognition. The findings suggest that eye blink rate is a useful proxy measure of dopamine levels in response to music. Notably, the “Winter” concerto had a higher blink rate than the other *Seasons*, indicating a more striking physiological response associated with its emotional content. However, the study found no evidence to suggest that arousal or music preference moderated the relationship between dopamine and music listening, although this aspect of the data should be treated with caution.

Dopamine’s role in the brain’s reward system and its regulation of motivation, pleasure, and reinforcement learning has been well-established in the literature. DA pathways activate when we anticipate a reward and again when we receive the reward, creating a cycle of desire and pleasure [52]. As a concrete example, this is why the sight and smell of foods can evoke a spontaneous urge to eat when we are not hungry [53]. Importantly, in disorders characterised by low DA levels, such as depression [54], we often turn to food for comfort or “self-medication” [55]. While pleasurable stimuli such as food can increase dopamine levels and reinforce behaviour, it is worthwhile to note that this is not the best method to enhance well-being, given the possible negative consequences. One could argue that music is a preferred source of dopamine-stimulating stimuli with no adverse outcomes. Several studies have demonstrated the release of dopamine in response to music, indicating its role in the pleasure and reward associated with music listening [12,35]. In contrast to other forms of self-medication for distressing affective states, music is seemingly devoid of adverse consequences, bolstering the argument that music occupies special status. In fact, music can alleviate depressive symptoms by helping patients label, communicate, and manage their emotions [9]. Furthermore, the anticipation of reward and reward value associated with listening to music can increase DA availability in the dorsal striatum and DA activity in the mesolimbic regions, respectively [35]. However, the specific effects of different types of music on dopamine levels have yielded mixed findings in the literature [40,42,56]. For example, sad music is associated with mind-wandering, while heroic music is associated with positive, motivational thoughts. Furthermore, emotions such as sadness and fear have been shown to correlate much more strongly with EBR than positively valenced emotions [33]. EBR is often used as a proxy measure of dopamine activity, as several studies have demonstrated a relationship between spontaneous eye blink rate and dopamine levels (for review, see [27]).

The influential work of Maffei and colleagues [33] is particularly relevant to the current study. Although the stimuli used were film clips rather than music, they demonstrated that clips eliciting negative emotions such as fear and sadness significantly correlated to increased EBR, while EBR relating to erotic clips, as well as those showing picturesque landscapes or depicting scenes which evoked compassion, negatively correlated with self-reported interest in the clips. It is worth highlighting that the researchers aimed to investigate not only the impact of the affective content of the stimuli but also the association between blink rate and allocation of attention to specific details of the stimuli. This aspect of their rationale bears some similarity to our own rationale, which speculates that blink rate is associated with attention allocation to internal versus external information processing. Therefore, it is important to keep in mind that an increase in eye blink rate may be attributable to both the valence of stimuli and the direction of attention towards internal and external worlds. The negative clips potentially induced mind-wandering or internal attention, whilst positive clips focused the viewers’ attention on the external stimuli. In the current study, whilst there were no correlations between EBR and major mode concertos (“Spring” and “Autumn”), EBR did significantly increase whilst listening to the minor mode concerto, “Winter”. These findings are consistent with the literature on music and dopamine, which suggests that negative emotions and sad music may be associated with increased dopamine activity [33,56]. Thus, it is possible that the melancholic, minor mode of the “Winter” concerto may have been responsible for the higher blink rate observed in the present study, as this piece of music evokes negative emotions in listeners. While our results align with the dopamine hypothesis, it is important to acknowledge the possibility that the alternative attention account may hold validity or serve as a partial explanatory factor.

Smilek et al. [37] concur. According to this view, high EBR may indicate a shift from external to internal processing rather than just a dopamine release. Accordingly, the blink rate can be influenced by external versus internal information processing, where attending to external information may lead to a decrease in blink rate, while engaging in introspection, self-reflection, and daydreaming may result in an increase in blink rate. Emotionally evocative music, such as sad music, may in particular lead to a decoupling of attention from external stimuli to internal thoughts and emotions. Walton [57] suggests that listeners empathise with emotionally relevant aspects of the music, such as tension, expressiveness, and imageability, and can project their thoughts and feelings onto the music. This could explain why sad music can have a cathartic effect, allowing listeners to safely re-experience and cope with negative emotions through the music. Garrido and Schubert [58] found that music empathy and absorption are associated with enjoyment of negative emotions in response to music listening. Enjoyment of sad music is related to aesthetic pleasure and mediated by feeling moved [59], and stronger emotional responses to sad music are associated with greater enjoyment of it [60]. Additionally, the aesthetic qualities of music can inhibit displeasure nodes in the brain, leading to pleasure responses to both happy and sad music [61]. Sad music is often enjoyed when it is aesthetically pleasing, non-threatening, and aids mood regulation, memory, and self-reflection [62].

With its minor mode and programmatic structure evoking imageability, the “Winter” movement may be a perfect candidate for this positive interpretation. The fast-paced *Allegro Non Molto* movement from “Winter” has been associated with high arousal and emotional valence [63], only second in arousal to the *Tempo Impetuoso D’estate* from “Summer”. Baltes et al. indicates that arousal is a stronger indicator of the distinctions between the *Seasons* than valence, with all 12 movements rated very positively. Thus, many of the assumptions of the previous literature, namely that the “Spring” movement is most engaging and pleasant, may need to be revisited. Familiarity may offer a better explanation, since the “Spring” movement is often rated as the most familiar to the general public as well as musicians, and previous research has indicated that reactivity in the reward pathway is stronger for familiar music [64]. Future studies on music and dopamine should account for these factors, in particular exploring whether affective reactivity, imageability, and empathy explain dopamine activation better than the positive valence of the music itself.

More generally, the existing literature on music and cognition may have underestimated the advantages of sad music, and will need to further investigate why, how, and in what circumstances sad emotions result in stronger dopaminergic reward. While individuals tend to prefer listening to mood-congruent music [65], the current results do not appear to support this. Instead of mood congruence or baseline preference for sad versus happy music, we may need to look deeper into the complexity of mood regulation. Saarikallio and Erkkilä [66] observed that adolescents use music in different ways depending on their mood before and the purpose. For example, while happy music is used for diversion and forgetting about negative feelings and events, sad or angry music is used for emotional discharge, such as releasing negative feelings. Looking at specific forms of sadness, *comforting sorrow* is related to perceptions of beauty, retrospection/memory, and introspection [67]. Thus, to understand the current study’s findings on the *Four Seasons*, and specifically “Winter”, we should also explore participants’ aesthetic appreciation and emotional reaction to the piece in greater detail, accounting for the complexity discussed above. A sad and thoughtful music piece such as “Winter” could be associated with varied psychological responses, from introspection and emotional release to imageability.

The measurement of arousal and music preference is crucial when investigating the potential impacts of music on cognition and brain function. Arousal levels can vary widely depending on the individual’s response to different musical stimuli, which can significantly impact cognitive performance and neural activity. Similarly, preference can influence an individual’s emotional response to music and may be related to the degree of neural activation observed. As previously noted, personality traits and mood predict enjoyment of music, such as extraversion and agreeableness, associated with stronger enjoyment of happy music, while depression and anger are associated with sad music [68]. Enjoyment also contributes to EEG reactivity, such as significant differences in alpha and theta waves for liked versus disliked musical excerpts [69]. In short, it may be the enjoyment of and preference for certain music pieces that result in stronger neural response rather than the baseline valence of the piece. This preference is also not static and is modulated by individual differences and mood.

While no consistent correlations between arousal and music preference and blink rate were observed in our study, it is possible that our method of measuring these variables was not sensitive enough to capture more subtle relationships. Schafer [70] suggests that a sample size of 85 is needed to observe a medium correlation of 0.30 at the desired power level of 0.80 [71], while the current study achieved a substantially smaller sample. The primary focus of this study was to examine the differences in blink rate across the concertos. Thus, the relationships and correlations between variables were of secondary concern and were primarily utilised to generate hypotheses for future experimental work. Nonetheless, it may be beneficial to revisit the observed correlations, particularly those with *p*-values just above 0.05, to address the power limitations of the study. Importantly, the current study used self-reported measures of arousal and mood, which could pose a methodological limitation. Previous research on emotional arousal in response to movies showed that physiological measures such as skin conductance and heart rate reveal differences in arousal not otherwise detected by self-report [72]. In short, further research is necessary before we can fully understand the relationship between arousal, music, and dopamine.

Our study also compared blink rates as a proxy measure of dopamine with another putative measure of dopamine. The P3a ERP component was identified in particular by Polich and colleagues (e.g., [48]). Proxy and indirect measures of brain function are vital for refining our theories of psychological phenomena when such processes are not directly observable. Interestingly, we did not find a significant correlation between these two measures. This may suggest that they are tapping into different aspects of dopamine function or are influenced by different factors. For example, while blink rate may reflect changes in dopamine release in the striatum, the P3a component may be more related to dopamine function in the prefrontal cortex (see [49] for a detailed examination of the neuropsychology and neuropharmacological origins of the P3a). The lack of correlation between these measures may reflect the complex and multifaceted nature of dopamine function. Therefore, although both are useful for indirect dopamine activity measurement in the brain and have similarities in where they have been applied for research purposes (e.g., Parkinson’s—blink rate [21]; P3a [73]), further work is warranted in this area. Although not directly associated with our discussion of dopamine, more recent work is beginning to systematically examine the brain responses coincident with eye blinks. Rather than ERPs, for example, Demiral et al. [74] used fMRI to track activity in brain networks, including the default mode network and associated blink responses. This approach offers a more comprehensive understanding of the brain’s involvement in blink phenomena and may contribute to our knowledge of cognitive and neural processes related to eye blinks.

In conclusion, our study has contributed to understanding the link between dopamine and blink rate in response to classical music. We found that the “Winter” concerto, in particular, had a pronounced effect on blink rate, possibly through differences in emotional reactivity and dopamine release. Notably, the programmatic qualities of the “Winter” concerto, such as its mournful melody, may drive affective processing and internal thought, feelings, and reflections, resulting in the observed impact on blink rate. Further research is needed to fully disentangle the underlying mechanisms, but our work demonstrates the utility of spontaneous blink rate to investigate how different musical qualities influence our physiology and internal information processing.

## Figures and Tables

**Table 1:** Blink rate (SD) in each of the concertos and the silence control condition across the three blocks.

	“Spring”	“Summer”	“Autumn”	“Winter”	Silent
**Block One**	33.6 (27.4)	35.5 (27.1)	36.8 (32.4)	40.8 (37.2)	33.1 (24.4)
**Block Two**	36.6 (27.6)	39.5 (32.7)	39.5 (30.8)	45.2 (31.5)	35.5 (29.6)
**Block Three**	33.9 (25.6)	38.2 (33.0)	43.5 (30.3)	49.9 (37.1)	34.2 (27.1)

## Data Availability

The research data are available at https://figshare.northumbria.ac.uk/ (10.25398/rd.northumbria.23264120).
